# Mr. Ward, on Opium

**Published:** 1803-04-01

**Authors:** M. Ward

**Affiliations:** Manchester


*March* 9, 1803.

---

### *To the Editors of the Medical and Physical Journal.*

Gentlemen,

I Flatter myself the request I am about to make to your Correspondents will not be thought unreasonable, especially if they will have the goodness to refer to Vol. vii. p. 192, of the Med. and Phys. Journal.

The favour I beg is, that they will be kind enough to suspend their opinions and remarks on my Papers on the Modus Operandi of Opium, until the whole of the evidence shall be laid before them.

Having proved that opium does *not* operate as a stimulant, which was what I first proposed (see Med. and Phys. Journal, Vol. vii. p. 128), I propose, 2dly, to prove, that it operates directly as a sedative; and, 3dly, to point out the practical advantages which this theory suggests. And after I shall have delivered my sentiments on these points, my first wish will be, that my observations and opinions may undergo a full and impartial scrutiny; and when this period arrives, it would give me pleasure to find they have attracted the attention of such of your readers as have employed a portion of their leisure time *in examining and comparing the experiments of different authors*, particularly those of Alston, Whytt, Monro, Johnstone, Alexander, and Wilson; the *study* of these authors being, in my humble opinion, a preliminary and necessary step, to enable any one to entertain just and proper notions of the properties and effects of the medicine.

They will doubtless see the necessity of entirely divesting ing themselves of every inclination in favour of ideas already imbibed; and, (instead of condemning my remarks indiscriminately, as two of your correspondents have done)* of pointing out *in what instances and respects* my theory is inconsistent with facts and experiments, my proofs are deficient, and my conclusions erroneous.

On a subject which has eluded the researches of so many men of first rate talents, there is reason to fear, many such instances will occur, but which may possibly admit of farther elucidation; and I shall, at all times, be happy to give every explanation in my power, but shall not consider myself bound to answer anonymous communications.

Some doubts having been expressed of the competency of Dr. Cullen's definitions, which I have hitherto taken as a guide, a necessity arises which obliges me to swerve from the proposed arrangement, until this question shall have been discussed.

You have very properly remarked, that no disputed point can be adjusted, while the disputants are employed on different subjects, or while the matter in question is not acknowledged by both parties to be the same.† You also doubt, (and your opinion will certainly have great weight) whether any advocates for the stimulant doctrine would admit of Dr. Cullen's definition of stimulants; but I persuade myself you will agree with me, that before it can be rejected, its insufficiency, and *inconsistency with the phenomena of muscular motion,* ought to be shewn, which has not yet been done; nor, (if mobility implies muscular action, and if to increase that be, as I imagine it is, a property essential to a stimulant) am I aware that it can be done.

Not that I think either this or Dr. Cullen's definition of sedatives, possessed of every attainable perfection; nor am I so invincibly attached to them as to object to their being changed, provided the conditions specified above (the reasonableness

---

* Med. and Phys. Journal, Vol. viii. p. 505. Vol. ix. p. 153—157.

† My own opinion is, that the question now at issue might have been decided long ago, had accurate definitions of the terms stimulants and sedatives been formed, *(and strictly adhered to in every dispute on the subject)* agreeably to such conclusions as are fairly deducible from the experiments of the three first authors quoted above; at all events, there can be no difficulty whatever in deciding it now that this branch of knowledge has been so much extended by the publication of many ingenious and accurate experiments.

sonableness of which is evident) are complied with. The latter, however, is more accurate and comprehensive than that of any author I am acquainted with; in as much as besides specifying the change produced in the system, it also informs us in what that change is supposed to consist, which is an essential circumstance.

But whether they are retained or given up, is, in my opinion, immaterial; only let their place be supplied by such as are accurate, and I shall be satisfied.

Were I indeed to refuse to submit to such regulations as are best adapted for the attainment of truth, either my motives or the equity of my cause might be justly suspected. And were I to succeed in endeavouring to suppress any evidence which might place the subject in a clearer point of view, my object would be defeated, which is, a clear and *final* decision of the question, founded upon just reasoning, accurate experiments, and established facts.

Another cause of my indifference as to what definitions it may be thought proper to adopt, is, a firm conviction of the impossibility of framing a just and accurate definition of the term stimulants, which will not exclude opium and alcohol from the list, or of sedatives, which *will* exclude them.

Undoubtedly, a definition might easily be formed so as apparently to include a particular substance, or even a class of substances, and which might appear *upon a cursory view* to be correct, on account of its adverting to some accidental or *secondary* change, frequently observed to result from their exhibition; but which, *on a close inspection,* would be found so exceedingly defective, as even to *exclude* that very substance, or class of substances, with a view to which it was composed.*

Of

* For instance, bleeding, both general and topical, uniformly operates as a sedative; and yet it frequently produces an *exhilarating effect*, and almost immediately increases the motions and powers of motions in many of the muscles of the body, which were before incapable of being moved; as, e. g. when it removes an acute pain in the side, or a spasmodic affection of the biliary ducts, intestines, &c. Now if opium be deemed a stimulant, because it frequently produces the effects just mentioned, bleeding might with equal propriety be deemed a stimulant; and so might any article in the materia medica, *when its operation is salutary*. But it may perhaps be said, bleeding does not *always* exhilarate, and seldom, if ever, produces this effect in health; nor does either opium or alcohol *always* exhilarate, and much seldomer have this effect *in health*, (considered independently of company,

Of this description, if I understood it aright, is Dr. Brown's definition of stimulants;* but what will certainly appear extraordinary, if not ridiculous, is, that it absolutely *excludes* those very substances from the class of stimulants, which he has placed *at the head of this class;* as I shall endeavour to demonstrate.

" Stimuli (says Dr. Brown, Elem. of Med. Vol. i. p. 6.) are either universal or local.

" The universal stimuli are the exciting powers, so acting upon the excitability, as *always* to produce some excitement *over the whole system.* And their appellation of universal is convenient, to distinguish them from the local.

" The local stimuli act only on the part to which they are applied; and do not, without previously producing an affection in it, affect the rest of the body."

Supposing these definitions to be correct, the doctrine inculcated in the following extract, is, in the highest degree, incorrect.

" Spirituous or vinous drink, in which the alcohol is always diluted, stimulates more quickly, and more readily, than seasoned food, and its stimulus is in proportion to the quantity of alcohol that it contains.

" But there are stimuli, which possess an operation as much quicker, and more powerful,† than these just now mentioned, and which are the agreeable and *proper ones* in health, as their operation is of shorter duration. To these the name of diffusible is to be given. They rank above *strong drink* in the following order:

Next to strong drink, and immediately above it, *stands* musk; above it, volatile alkali; higher than this, æther; and the highest of all, as far as experiments have yet reflected light upon the subject, is opium." (Brown's Elem. Vol. i. p. 103—4).

Unfortunately however for the Brunonian theory, instead of these articles *always* producing some excitement over

---

company, conversation, &c. *which are in themselves highly exhilarating)* than is commonly imagined; and even when these are joined, it is not uncommon to hear persons *not debilitated by intemperance,* complain, that *wine,* as well as spirits, generally induce dulness and languor, especially after taking food; and that they are *stronger* as well as more alert, when they abstain than when they indulge in their use.

* I do not mean to say that it was composed with a view to suit a particular class of substances.

† " Than that of the articles of diet."

*over the whole system,* they NEVER have this effect;* at least I have not met with any experiment (and I have examined a great number) where this appeared to be the fact. On the contrary, I find they *always* produce *a diminution of the excitement in the fibres and vessels of the part to which they are applied,* (whenever it is so situated as to admit of inspection :) *and if the whole system does become affected in consequence of their application,* THE EXCITEMENT IS ALWAYS UNIVERSALLY DIMINISHED.

Can a stronger proof of the fallacy and imbecility of the Brunonian System be required?

The experiments from which my information has been chiefly derived are those of Drs. Alston, Whytt, Monro, Bard, Fontana, Johnstone, Alexander, Wilson, and Crumpe.†

Particular proofs will of course be required in support of my assertions; and these shall be as numerous as my opponents could wish, or, at any rate, more so than they could reasonably expect.‡

I shall begin with such experiments as had been published long before Dr. Brown's opinions were made public; and which are more than sufficient, one would have imagined, to have prevented such unwarranted assertions from being made; or at least, from gaining assent.

Dr. Alston's experiments with opium on frogs, stand in direct opposition to Dr. Brown's assertions; and they are the more satisfactory, as they were frequently repeated, and always with the same appearances and event.

So far is Dr. A. from making use of any expression which could justify the supposition of its producing some excitement

---

* Musk is here excepted, as I do not know that any experiments have been made to ascertain its mod. op.

† To which may be added two experiments I made with opium applied externally, and which afford a strong confirmation of the *directly sedative properties* of opium. See Med. and Phys. Journal, Vol. VII. p. 135—7, and 355—8.

‡ Though, indeed, the onus probandi seems to rest with them, their leader having made a string of assertions, which at present stand unsupported by proofs; it is therefore incumbent upon them to supply the deficiency, and to prove, *by clear and decisive experiments,* that alcohol, volatile alkali, æther, and opium, *always* produce some excitement *over the whole system.* If they fail in this respect, they must of necessity either abandon the Brunonian definition of stimulants, or the doctrine founded upon it.

This appears to be the most regular mode of proceeding; but, probably, the informality will be excused, and it may be the means of saving time, if I proceed immediately with my proofs.

excitement *over the whole system,* that he expressly says, that Mr. Fullarton and himself *very distinctly saw,* (in the frog's foot) A SURPRIZING DIMINUTION OF THE BLOOD'S VELOCITY; *for,* says he, *it did not move half so swiftly as it uses to do in these creatures.* We alternately looked at it *again and again, and in less than half an hour saw the velocity of the blood* GRADUALLY INCREASE, *the uneasy frog* RECOVER ITS WONTED VIGOUR, AND THE BLOOD ITS COMMON CELERITY; upon which we took out the paddock, put it in a bason of clean water, and allowed it half an hour to refresh itself; then gave it another dose of opium, fixed it to the microscope *with all expedition,* and viewed it as before; *the blood then moved yet slower than it did the first time,* AND ITS VELOCITY GRADUALLY DECREASING, *at length it stagnated, first in the smaller, then in the larger vessels, and in about a quarter of an hour the animal expired.**

Nor is it possible to conceive experiments could be more hostile than those of Drs. Whytt and Monro are to the opinions of Dr. Brown; and their remarks and inferences, which are in general consistent, clear, and instructive, are equally so. Not that either Dr. W. or Dr. M. appears to have had any particular theory in view; but have merely given the result of their experiments, with such observations and conclusions as they suggested.†

The same remarks are applicable to Dr. Bard's Dissert. de Viribus Opii, published in 1767. His intention however seems to have been, to prove that opium acts as a sedative; but this does not invalidate his *facts,* or render his Thesis, (which is written with great candour,) less deserving of attention.

I could with great pleasure make many extracts from each of these works, which would greatly strengthen my arguments, but not without anticipating the evidence which will be brought forward, with more propriety, when I come to consider the second proposition, stated at the beginning of this letter.‡

---

* Ed. Med. Ess. Vol. v. part 1, p. 153—6. See also Med. and Phys. Journ. Vol. VII. p. 359—360.

† The experiments of Dr. Monro are not confined to opium, but also include alcohol and camphire. I shall take this opportunity just to intimate a wish, that he had avoided stiling camphire an essential oil, as its medicinal properties are, in many respects, so *opposite* to those of essential oils, strictly so called.

‡ Some of Dr. Monro's experiments, and one of Dr. Whytt's, have been already quoted. See Med. and Phys. Journ. Vol. VII. p. 342—344—350—354. Vol. VIII. p. 343—5.

But I cannot refrain from copying a few of Dr. Whytt's *general conclusions.*

"From the preceding experiments, (says Dr. Whytt) we may, I think, fairly draw the following conclusions.

"Opium applied to the stomach, guts, cavity of the abdomen, thorax, and abdominal muscles, *soon lessens,* and after some time *entirely destroys* ALL FEELING AND POWER OF MOTION, NOT ONLY IN THE PARTS TO WHICH IT IS APPLIED BUT THROUGH THE WHOLE BODY.

"It remains, therefore, that opium, by affecting the extremities of the nerves of the parts to which it is applied, does, by means of their connexion and sympathy with the brain and spinal marrow, *destroy or prevent,* THROUGH THE WHOLE NERVOUS SYSTEM, THE OPERATION OF THAT POWER UPON WHICH DEPENDS SENSATION AND MOTION IN THE BODIES OF ANIMALS.*

"Opium *does not only destroy the moving power of the muscles of animals,* by intercepting the influence of the brain and spinal marrow, *but also by unfitting the muscular fibres themselves, or the nervous powers lodged in them, for performing its office;* otherwise a solution of opium, when applied to the abdominal muscles or viscera of a frog, would not put a stop to the heart's motion sooner, or indeed so soon, as decollation and the destruction of the spinal marrow. (No. 4 and 5 compared with No. 8 and 10.) Opium therefore does not produce its effects, *solely,* by putting a stop to the function of the brain and spinal marrow, *but its influence reaches to the fibres of the muscles themselves, or to the extremities of the nervous filaments which terminate in them.*

"When I say the influence of opium reaches to the nervous filaments which terminate in the muscular fibres, it is not meant, that any effluvia or subtle parts of the opium are transmitted to them ('see *n* and *o* above') but that it destroys their powers, by means of that sympathy which they have, through the brain or spinal marrow, with the nerves to which the opium is immediately applied.

* Dr. Wilson, in his Essay on Opium, p. 12, makes the following striking remark on this passage:—"I cannot, however, conclude my observations on this subject, derived from the labours of Dr. Whytt, without quoting from his paper the following passage, which, as far as I can judge, *either from my own experiments, or from those of others, contains one of the justest, and, at the same time, one of the most important observations concerning the action of opium on living animals, I have any where met with.*"

"In animals which have got a large dose of opium, the veins, especially those of the membranes of the brain, are observed to be much swelled; whence it has been thought, that opium produces its effects in the bodies of animals, partly, at least, by rarefying the blood and compressing the brain; *but this distension of the veins seems to be no more than a consequence of the very slow motion of the blood through the heart, on account of the insensibility with which this organ is affected.**

"Since opium soon puts a stop to the vital motions of animals, which yet continue in time of sleep with little or no diminution of their vigour; since it often eases pain without bringing on sleep; and since, by its topical action on the heart, it destroys the motion of this organ after all communication between it and the origin of the nerves is cut off; it follows, that the effects of opium are not owing, as some have thought, to its producing sleep; *on the contrary, the sleep which it occasions, seems to be only a consequence of its impairing the sensibility of the whole nervous system.*

"The other effects of opium may be also deduced from the same cause, *particularly its restraining all evacuations that are owing to an usual irritation of the parts of the body, and at the same time promoting those natural secretions which have been diminished or stopt by spasmodic strictures of the vessels, from some uncommon* STIMULUS *affecting them.*"

"Lastly, does not opium kill animals *by rendering their several organs wholly* INSENSIBLE OF THE STIMULI, WHICH ARE DESTINED BY NATURE TO EXCITE THEM INTO ACTION; *whence, not only a stop is put to the peristaltic motion of the guts, and to the propulsion of the chyle,*† *but the fluids also begin to stagnate, first in the smaller and afterwards in the larger vessels;*‡ *while the heart becoming* GRADUALLY

* "In frogs into whose stomach and guts I had injected a solution of opium, I not only found the heart's auricle, but also the great veins leading to it, much distended with blood." Vid. Essay on Vital Motions, &c. p. 371—2.

† Here Dr. Whytt quotes the well known experiment of Dr. K. Boerhaave, which has been already inserted in the Med. and Phys. Journal, Vol. VII. p. 498.

‡ "This my worthy colleague, Dr. Alston, observed with a microscope in frogs into whose stomach he had conveyed a few drops of a solution of opium in water. Vid. Med. Ess. Vol. v. part 1, art. 12. And indeed the great distension of the heart and its auricle in frogs killed with opium (No. 5, compared

GRADUALLY *less sensible of the stimulus of the blood with which it is distended, contracts more feebly and at greater intervals, till at last it ceases from motion altogether."* Ess. and Obs. P. and L. vol. ii. p. 301, 302, 309, 310, 313—316.

Let any impartial man peruse the experiments from which these conclusions, and many others equally decisive, are drawn, and also the experiments and remarks of Drs. Alston, Monro, and Bard; and then ask himself, whether Dr. Brown was justifiable in asserting, that opium, *as far as experiments have yet reflected light upon the subject,* can be said to occupy the highest place, *(or any place at all)* in the class of stimulants? If it be possible for him to entertain a doubt, let him then peruse the experiments, &c. of Fontana, but particularly those of Drs. Johnstone, Alexander, and Wilson; (he may also peruse the experiments and arguments of Dr. Crumpe, with my comments upon them,) and it would in my opinion be a great reflection upon his understanding, to suppose that he could still think the opinions of Dr. Brown, on this subject, tenable.

That assertions so destitute of proofs should have gained credit almost universally, would be scarcely credible, were not the fact well known; nor does it afford a strong proof of the sagacity of his approving cotemporaries.

Was it not incumbent upon Dr. Brown to have proved, that the experiments of Dr. Whytt, &c. were false, or that their conclusions were unwarranted by their experiments, before he had ventured to bring forward assertions in direct opposition to them? This was, I think, the least that could have been expected.

The experiments of the five remaining authors quoted above, have been published since Dr. Brown's Elements of Medicine; but though the evidence they afford of the *directly sedative properties* of alcohol, volatile alkali, æther, and opium, is as positive and decisive as could be wished or even imagined, yet the number of converts to the Bruno-

---

compared with No. 3, 6, and 10 above) indicates a more than ordinary resistance to the blood's motion in the arteries, as well as a less degree of irritability in the heart. Further, is not the slow, full pulse, and dry parched mouth, in those who have got an over-dose of opium, owing, *partly to the slower motion of the fluids in the small arteries and secretory vessels of the glands?* Though it must be confessed, that the dryness of the mouth may be in some measure owing to the perspiration being greatly increased by the opium."

nian system seems to have increased, and to be increasing, in a manner truly alarming and unaccountable; and I fear I have no great reason to flatter myself that my endeavours to stem the torrent will be crowned with success; but I will do my utmost, in hopes that truth, on which ever side it may prove to be, may ultimately prevail.

I have already had occasion to make extracts from the experiments of each of these authors excepting Dr. Johnstone's,* (particularly from those of Dr. Crumpe,†) and these I consider as so many indisputable proofs in support of my arguments, but not more strong than many others which still remain to be considered, though some are more comprehensive than others, and demonstrate more clearly the directly sedative properties of the articles which Dr. Brown has placed at the head of the class of stimulants,) but which, for the reasons already alledged, I shall decline bringing forward at present. But that I may at the same time fulfil the promise made to the advocates of the stimulant doctrine, I shall refer them to the experiments of *all* the different authors mentioned above, which I have already said I consider as so many undeniable proofs in my favour; from which they are at liberty to select such as they may deem best adapted to prove, that opium, alcohol, &c. *always produce some excitement over the whole system.*

Not that I wish to limit them even to these, or to prescribe any boundary, provided such experiments as are of a doubtful nature, or which the experiments of other writers have shewn to be inaccurate, are avoided. And yet, with all these advantages, I will venture to say, they will not find it an easy matter to produce *one* such instance.

I shall conclude with a few general Observations on the term Stimulants.

I have often been surprised, that a term so plain, precise, and definite in its signification, should have been so strangely perverted from its true meaning, as to have been applied indiscriminately to substances whose nature and mode of operating are totally dissimilar; as much so as those of astringents and cathartics, acids and alkalis, fire and water, &c. &c. Whatever may have been the original source of the error, it has been the means of introducing great

---

* Vid. Med. and Phys. Journal, Vol. VII. p. 344—6. 351—2. Vol. IX. p. 45—53.

† Idem. No. 36, 38, 40, 44, 45, 47.

great confusion into the theory and practice of medicine; insomuch, that, at present, no phrase can be more vague and indefinite in its meaning, than that of "a stimulant plan of treatment."

Instead of this phrase being confined, as I think it ought to be, to such things as *increase the contractility*,* *tone, and vigour of the muscular fibres of the body*; † it seems, in the common acceptation of the phrase, to signify, the frequent exhibition, *in large doses*, of such medicines as *always immediately diminish or destroy the action or contractility of the muscular fibres of the part to which they are applied*; ‡ and if they are applied to parts endowed with involuntary motion (in some animals and under certain circumstances hereafter to be noticed) such as the stomach, the intestines, the heart, or large blood vessels, (the nervous system being entire) *all the muscles of the body are found, when examined immediately after death, to be totally insensible to every kind and degree of stimulus.* Now, if this be their mode of operating, and that it is, experiments abundantly prove, it is as absurd to stile them stimulants, as it would be to call astringents cathartics, alkalis acids, or black white. And yet, strange as it must appear, several writers of eminence have fallen into this mistake.|| And what adds to this apparent inconsistency is,

---

* I am much pleased with Dr. Alexander's substitution of the word contractility for irritability.

"Per contractilitatem, eam dotem intelligi volo, quæ irritabilitas nominatur, ab Hallero aliisque fibra musculari insitam esse; a Whytt, Monro, et iis quibus nervorum doctrina maxime arridet, a nervis in fibram muscularem diductam, existimatam. Verbum contractilitas mihi præ aliis placuit, quia pericula fere semper in fibras musculares instituta sunt. Terminus est etiam qui vim earum contrahendi magis accurate exprimit quam irritabilitas, quippe quæ latiorem significationem habet, et, juxta quorundam sententiam, proprietas est toti corpori vivo communis, vel, ut aliis verbis utamur, vitæ fons et origo est." Vid. Dissert. p. 113.

† Such as decoctions or infusions of bark, with cinnamon or other aromatics; of orange peel with gentian or cinnamon; preparations of myrrh, steel, and guaiacum; moderate quantities of animal food, with spices, mustard or horse radish; and small quantities of wine; together with country air, and moderate exercise on horseback; bathing; frictions either dry or medicated; cheerful company, conversation, &c. &c.

‡ Namely, alcohol, volatile alkali, æther and opium, to which may be added camphire, which seems, from the experiments of Dr. Monro, and the remarks of Dr. Cullen, to operate upon the same general principles.

|| Vid. Brown's Elements of Medicine, Darwin's Zoonomia, Alexander's Dissertation on Opium, Beddoes's Hygeia, &c. &c.

is, that one of them (Dr. Darwin) seems (if we may judge from his definition of *incitantia*, and of the term *stimulus*, as

---

In support of what has been advanced, I shall take the liberty of copying the inferences which Dr. Alexander draws from his experiments:

" Conclusio."

" Partibus corporis, in quas opium actionem suam exercet, pro virili indagatis, ea quæ ab experimentis nostris rite deducenda, ad argumentum nostrum immediate pertinent, repetere haud abs re fore judico.

" 1mo. Opium fibræ musculari admotum contractilitatem ejus nunquam non consumit. Vid. Exp. 1, 4, 7, 8, 11, 12, 14, &c.

" 2do. Spiritus vini, *et alii stimuli valentiores*, eosdem effectus præstant. Vid. Exp. 2, 5, 9, &c.

" 3tio. Opium, eadem qua animal, admotum, contractilitatem fibræ temperatura inferiore, citius, consumit. Compara Exp. 4, cum 11, 12.

" 4to. Opium applicatum cordibus animalium, quæ motus voluntarii facultatem et vitam, brevi tempore post thoracem apertum, amittunt, quamvis contractilitas in systemate pene integra restet, vim tamen contractilem musculi totius, cujus pars actioni ejus subdita fuerat, solum consumit, dum reliquæ corporis partes, ut antea, contractilitatem suam conservant. Vid. Exp. 14, a et b.

" 5to. Effectus opii, admoti cordibus animalium quæ corde privata, per tempus aliquod vivunt, et motus voluntarios efficere perstant, per totum corpus dispertiuntur. Vid. Appendix B. Exp. 1, 2.

" 6to. Opium in ventriculum, vel sub cute, eosdem fere edit effectus ac in cor injectum, scilicet, mortem infert, contractilitatem delet. Vid. Exp. 20, 21, 47, 48, &c. &c.

" Sed hoc notatu dignum est,

" 7mo. Quando sat opii ad animal intermiendum musculis voluntariis admovetur, musculi involuntarii contractilitatem suam adhuc conservant. Vid. Exp. 47, 48, 52, 59, &c. &c.

" 8vo. Musculis involuntariis adhibitum, non modo eorum, sed etiam musculorum voluntariorum, contractilitatem abolet. Vid. Appendix D. Exp. 1, 2.

" 9no. Opium partes corporis longe remotas non ideo afficit, quod musculi inter se nectuntur, nec quod contractilitas, natura, indivisa est. Vid. Exp. 42, 43.

" 10mo. Nullum talem fluidorum conditionis mutationem infert, qualis ad mortem partium remotarum inducendam, vel ad contractilitatem harum perdendam, valeret. Vid. Exp. 15, 16.

" 11mo. Opium corpori applicatum ea quantitate resorberi posse, quæ animal enecaret, minime constat. Vid. Exp. 47, 48, 52, 59, &c. &c.

" 12mo. Et res haud ita se habeat necesse, quia opium eandem conditionem absorbentium, ac cæterarum partium musculis instructarum, movere videtur. Vid. Exp. 51.

" 13tio. Vim suam in cerebrum et nervos exhibet. Vid. Exp. 29, 30, 31, &c.

" 14to. Nexu inter nervos intercluso, effectus ejus haud palam fiunt ultra fines nervorum istius partis, cui immediate admovetur. Vid. Exp. 42, 43, 44.

" 15to. Systemate nervoso incolumi, omnes corporis partes sub imperio nervorum afficit. Vid. Exp. 35, 36, 38, 39.

" 16to. Quamobrem nervis debemus, quod opium effectus suos longe lateque diffundit. Alex. de Opio, p. 108—111."

as well as of the different terms employed in those definitions, and the observations connected with them) to consider *contraction in the muscular fibres as the immediate and necessary consequence* of the application of stimulants.

Dr. Darwin's definitions, above alluded to, are as follow:

"Those things, which *increase the exertions* of all the irritative motions, are termed incitantia." Zoon. vol. ii. p. 678.

"By the word stimulus is not only meant the application of external bodies to our organs of sense and muscular fibres, which *excites into action the sensorial power termed irritation*; but also pleasure or pain, when they *excite into action* the sensorial power termed sensation; and desire or aversion, when they *excite into action the power of volition*; and, lastly, the *fibrous contractions*, which precede association; as is further explained in Sect. 12. 2. 1."

"A certain quantity of stimulus produces irritation, which is *an exertion of the spirit of animation exciting the fibres into contraction.*

"There are three circumstances to be attended to in the production of animal motions. 1st. The stimulus. 2d. The sensorial power. 3d. *The contractile fibre.*— 1st. A stimulus, external to the organ, originally induces into action the sensorial faculty termed irritation; *this produces the contraction of the fibres*, which, if it be perceived at all, introduces pleasure or pain; which in their active state are termed sensation; which is another sensorial faculty, *and occasionally produces contraction of the fibres*; this pleasure or pain is therefore to be considered as another stimulus, which may either act alone or in conjunction with the former faculty of the sensorium termed irritation. This new stimulus of pleasure or pain either induces into action the sensorial faculty termed sensation, *which then produces the contraction of the fibres*; or it introduces desire or aversion, which excite into action another sensorial faculty termed volition, and may therefore be considered as another stimulus, which either alone, or in conjunction with one or both of the two former faculties of the sensorium, *produces the contraction of animal fibres.* There is another sensorial power, that of association, which perpetually, in conjunction with one or more of the above, and frequently singly, *produces the contraction of animal fibres*, and which is itself excited into action by *the previous motions of contracting fibres, &c. &c.*" Zoon. vol. i. p. 13, 30, 73, 74, &c.

Upon

Upon the whole, if the above observations are just, the obvious inference is, that an improved definition of stimulants might and ought to be formed in perfect conformity thereto, to be strictly adhered to on all occasions, and to the exclusion of every other.

Some such plan as this can alone, I conceive, by bringing us back to just principles, remove the confusion and uncertainty which at present prevail.

What would be thought of the following?

Stimulants are such substances, or powers, as *increase* the contractility of the muscular fibres locally or universally.

Sedatives are such substances, or powers, as *diminish* the contractility of the muscular fibres, locally or universally.

I am aware that the first of these definitions would exclude alcohol, volatile alkali, æther, opium, camphire, and several other medicines, at present considered as stimulants, from the list; and for that reason, that it will not be perfectly agreeable to *all* parties. But if this be the only, or the *principal* objection that can be urged against it, it ought not, and I trust will not, prevent either this, or some other more perfect definition from being adopted; this being an affair of too much moment to allow private or partial considerations to have any weight in the decision. I am, &c.

*Manchester, March* 9, 1803. M. WARD.

P. S. The request with which the above letter commences, precludes me from replying to Mr. Hill's communication, inserted in No. XLVIII. I shall therefore only observe, that my sole view in selecting the Quotation to which Mr. H. alludes, was merely to sanction *the publication of the facts* which had occurred to me, and that my choice (indeed it was suggested to me by a friend for whom I entertain the highest esteem) was not at all directed by any opinion I had formed of the modus operandi of opium, (for I confess I had not then paid sufficient attention to the subject, to enable me to make up my mind upon it) much less of its manner of operating in the disease treated of by Mr. Pott.——It will not, I hope, be deemed inconsistent with the tenor of my request, to desire Mr. Hill will have the goodness to point out, through the medium of the Medical Journal, in which of my Papers I have spoken of opium as a *tonic stimulant,* or have attributed *opposite* qualities to it.

